# Pluripotent Cell Models for Gonadal Research

**DOI:** 10.3390/ijms20215495

**Published:** 2019-11-04

**Authors:** Daniel Rodríguez Gutiérrez, Anna Biason-Lauber

**Affiliations:** Endocrinology Division, Department of Endocrinology, Metabolism and Cardiovascular System, Section of Medicine, University of Fribourg, 1700 Fribourg, Switzerland; daniel.rodriguez@unifr.ch

**Keywords:** induced pluripotent stem cells, iPSCs, DSD, cell model, sertoli cell, granulosa cells, germ cells, cellular reprogramming, patient-specific model

## Abstract

Sex development is a complex process involving many genes and hormones. Defects in this process lead to Differences of Sex Development (DSD), a group of heterogeneous conditions not as rare as previously thought. Part of the obstacles in proper management of these patients is due to an incomplete understanding of the genetics programs and molecular pathways involved in sex development and DSD. Several challenges delay progress and the lack of a proper model system for the single patient severely hinders advances in understanding these diseases. The revolutionary techniques of cellular reprogramming and guided in vitro differentiation allow us now to exploit the versatility of induced pluripotent stem cells to create alternatives models for DSD, ideally on a patient-specific personalized basis.

## 1. Introduction

The normal gonadal development in humans requires the participation of two types of cells: germ cells and somatic support cells. The germ cells are the precursors that will originate the corresponding gametes, oocytes in females and sperm cells in males. The progressive transformation of primordial germ cells into gametes cannot be accomplished without the tight control of the somatic support cells that surround them.

**Figure 1:**
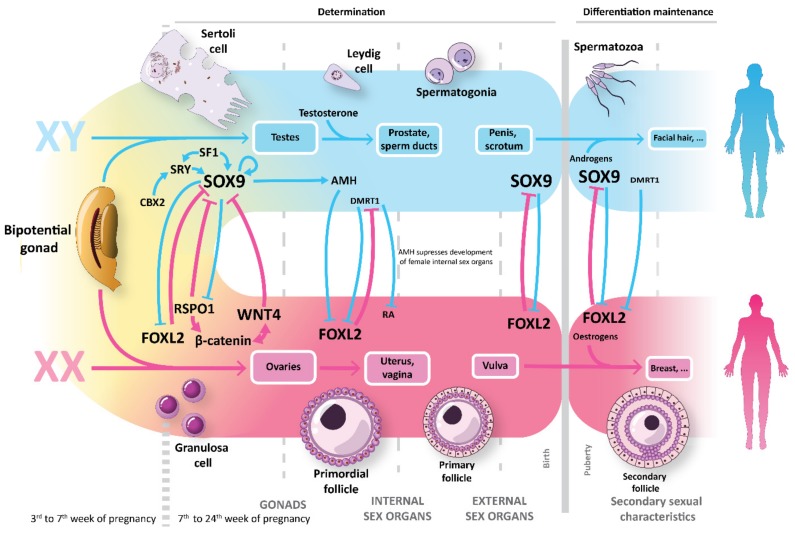
Human sex determination and differentiation: Gonadal somatic supporters, Sertoli cells and granulosa cells, play a central role in sexual development, orchestrating the differentiation of the other gonadal cells and secreting SOX9 and forkhead box L2 (FOXL2). Both factors counteract each other in a delicate equilibrium and its expression must be maintained through different stages of development to keep the final sexual faith into males or females respectively.

In males, this supportive role is taken up by Sertoli cells (SCs). SCs constitute the structural component of the seminiferous tubule, creating the appropriate niche for the germ cells isolating them from the soma thanks to the establishment of the blood–testis barrier (BTB). Yet, the role of SCs is not limited to that. They produce the sex-determining region Y-box 9 (SOX9), a key factor during male sex development, and they also perform a precise control on the cellular transformation of spermatogonial stem cells into mature spermatozoa through the secretion of anti-mullerian hormone (AMH) (Figure 1). It is because of this control over the germ cells that they are often referred as “mother” or “nurse” cells [1]. The steroidogenic production of testosterone is carried out by Leydig cells located in the interstitial tissue between seminiferous tubules [2]. At puberty, SCs mature reducing dramatically the expression of AMH and their proliferation and motility, gaining the ability to support spermatogenesis [3,4].

In females, the supporting role is taken up by the granulosa cells (GCs), also known as follicular cells. In contrast to their male counterpart, one can differentiate between several groups of granulosa cells with specific roles in the control of the oocyte environment. Localized tightly surrounding the primordial oocytes, a group of GCs known as cumulus cells (CCs) control the differentiation and maturation of the oocyte through cell-to-cell interaction (via gap junctions) and paracrine action.

A major player in the development and maintenance of the female phenotype is wnt family member 4 (WNT4): this secreted protein is almost exclusively expressed by GCs during early ovarian development, and also regulates the formation of Müllerian ducts. WNT4 counteracts the effects of fibroblast growth factor 9 (FGF9) and SOX9 in a very delicate equilibrium, with WNT4 turning the balance into ovary development and FGF9 and SOX9 into the testis side. The CCs also produce AMH, whose correlation with follicular aging was well stablished [5].

A second type of GCs known as mural granulosa cells (MCs) rest on a basal lamina and separate the oocyte environment form the vascularized theca interna, creating a blood–follicle barrier. The mural granulosa cell produces a much lower amount of AMH than cumulus cells and its endocrine function is oriented to the production of steroidogenic enzymes like Cytochrome P450 aromatase and the conversion of androgens from the theca cells (androstenedione and testosterone) into estrogens like estradiol [6].

Both populations of GCs show expression of the forkhead box protein L2 (FOXL2) which counteracts the action of SOX9 in female sex development [7,8]. The expression of FOXL2 is indeed observed very early in ovarian development, in the cellular progenitors of GCs. FOXL2 acts over transcriptional targets of various cellular pathways [9]. Studies in mice and goats associated FOXL2 with the repression of SOX9-guided testis determination [10,11]. In a complimentary manner to the FOXL2 signaling pathway, WNT4, β-catenin (CTNNB1) and R-spondin 1 (RSPO1) act during ovarian differentiation to inhibit SOX9 effect on transdifferentiation of pre-granulosa cells (pre-GCs) into SCs after sex determination in mice [12]. Still many things remain unknown about the role of both FOXL2 and RSPO1 in humans but, interestingly, RSPO1 expression had been be detected in human undifferentiated gonads of both sexes but it is upregulated only in the ovary during critical stages of early gonad development (6–9 weeks post fertilization) [13]. Apart from FOXL2, CCs and MCs show marked differences in the expression and regulation of receptors for the luteinizing hormone (LH) and follicle-stimulating hormone (FSH), central elements in the ovarian follicle development [14]. In females, the communication between germ cells and somatic supporters occurs in both directions, having oocyte also control cumulus cells via growth differentiation factor 9 (GDF9), oocyte secreted factors (OSFs) or bone morphogenic protein 15 (BMP15) [15,16].

Alterations of the gonadal germ and/or somatic cells at a genetic or structural level during sexual development often lead to differences/disorders of sex development (DSD) in humans and many different species [17,18,19]. The term DSD involves a group of complex conditions that can affect chromosomal, gonadal, and/or phenotypical sex. These diseases are heterogeneous and not as rare as previously thought: it is estimated that genital anomalies occur in 1:4500–5500 births [20]. They compromise not only internal and external genitalia formation, but also affect fertility to various degrees [21]. AMH expression was observed to be altered in 46,XY DSD conditions like complete and partial gonadal dysgenesis, and ovotesticular dysgenesis among others [22,23]. In fact, AMH levels have become a highly reliable indicator of the existence of testicular tissue in prepubertal patients, while undetectable levels suggest testicular dysgenesis. 

Based on results from affected mouse gonads, under loss of FOXL2, SOX9 expression is de-repressed in GCs, which transdifferentiate into SCs. In humans, heterozygous FOXL2 disruption was associated with premature ovarian failure (POF) [24] but homozygous FOXL2 deletion patients have not been reported so far. WNT4 mutations have been associated with Müllerian-duct regression and virilization in 46,XX patients [17] and some cases of 46,XX testicular and ovotesticular DSD are linked to mutations of the RSPO1 gene [25,26]. All these hints suggest that the genes responsible for GCs differentiation also have a high impact on ovarian development.

Consequently, the study of the actions of germ cells and their somatic supporters at a cellular level has been revealed as essential to understand the complex mechanisms underlying the different DSD. In this review, we will analyze the different approaches to modeling DSD gonadal cells and the doors that new induced pluripotent stem cells (iPSCs) technology has recently opened for DSD research.

## 2. Finding a Perfect Match: The Lack of Right Models for DSD Patients

Due to the high complexity and the great variety of DSDs, the most useful approach would be the study of cells extracted directly from the patient. The first obstacle that this methodology faces is the fact that in some severe cases of DSD with complete gonadal dysgenesis, the patient lacks those types of cells. This limitation cannot be overcome with any of the currently available cellular models (see below), and led to the search of new approaches, ultimately leading to personalized cell modeling. Even when gonadal somatic supporter cells are present, their very limited number significantly complicates the establishment of primary cultures. Animal models and cell lines derived from gonadal tumors have so far been the main alternatives to study the mechanism of disease that lead to DSD. Such models, though, show important differences due to their tumoral origin and the fact that the sex development pathway is not particularly well conserved among species.

### 2.1. Sertoli Cell Models

Most of the in vitro studies on SCs development were performed in immature rodent testes. The first SC line established, the mouse TM4 cell line, was generated by passing cultures of immature mouse SCs over many generations to generate an immortal line [27]. Other mouse SC-models like 15P-1 cells, where immortalized from testis of transgenic mice expressing large T protein of polyomavirus [28]. Although useful to understand common characteristics of mammalian SCs, murine models have been demonstrated to differ from humans regarding sex determination and development [29,30,31]. Murine SCs have also shown little proliferative ability in vitro, leading to the thought that mammalian SCs do not divide postpuberty. This hypothesis has been recently challenged when studies with SCs isolated from cadaveric testes [32] or donors undergoing bilateral castration [33] demonstrated the ability of human SCs to proliferate when cultured in vitro. Thus, primary human Sertoli cell lines known as HSerCs are now commercially available. Techniques like differential plating seems to improve the viability of isolated SCs [34] but it is very important to remind that, when the focus of the study is the role of SCs in male development, a reliable source of immature SCs from prepubertal patients is essential. Adult patients lack those immature cells and testicular biopsies from very young patients pose important ethical questions.

An alternative that overcomes the problem of in vitro culture of SCs is the use of human cancer cell sources. One of these cell lines is represented by NT2d1 cells derived from pluripotent clonal cells obtained from testicular tumors [35]. NT2d1 cells were extensively used for SCs studies since they express most of genes involved in sex determination [36]. However, we have recently observed a very different transcriptome pattern between NT2d1 cell and primary human SCs when we observed genes related with SC differentiation and maturation [37]. Those findings suggest that their carcinogenic origin partially altered their SC characteristics, and renders these cells less than an ideal model for SCs.

### 2.2. Granulosa Cell Models

Human GCs are often recovered from women undergoing in vitro fertilization (IVF) after ovarian stimulation and ovulation induction. GCs are isolated from follicle fluid during oocyte retrieval procedures. It is important to remark that, following ovulation, GCs undergo a process of luteinization which involves structural and genomic changes that lead to the terminal differentiation of follicular cells with increased progesterone production [38]. The differentiation of granulosa cells into luteinized cells has effects on intracellular signaling and cell cycle regulation. As mature SCs, luteinized GCs stop their proliferation, which makes the long-term cultivation of GCs primary cells extremely challenging [39].

The initial studies in this direction by Smith et al. [40] and Byong-Lyul et al. [41] confirmed a transformation of the harvested population of GC, observing two different populations in vitro. Some luteinization processes seem to be initiated in these cells but they never achieved the lutein levels observed in situ. It became clear that GCs faith is extremely dependent on its microenvironment. Bruckova et al. [42] described the negative effect of high concentrations of fetal calf serum (FCS) in culture medium, promoting the apparition of a very heterogeneous population that degenerate after a short period (18 days) in culture. The presence of LH acts as a trigger for the luteinization process reducing GCs proliferation while FSH induces a series of mitotic divisions. In a pioneer study the same group [39] highlighted the importance of the follicle fluid in addition to growth factors like fibroblast growth factor basic (bFGF) and epidermal growth factor (EGF) to maintain the proliferative potential of GCs primary cultures. This work shed some light on the preservation and culture of GCs from patients whose gonads have suffered alteration but are not completely absent while it is still unclear if altered GCs will require a different microenvironment to keep their mitotic potential.

In the same fashion as SC, researchers resorted also to cell lines derived from carcinogenic tissues. KGN as well as COV434 cells were obtained from human ovarian granulosa cell tumors [43,44]. KGN cells became the most common cellular model used for the study of human granulosa cells. Although KGN have great potential for steroidogenic studies and their proliferation and immortality make them an easy model to work with in the laboratory, characterization analyses showed that KGN have a mutated form of FOXL2 related to the loss of apoptosis induction mechanism in granulosa tumors. This mutation was not present in any of the other cell models analyzed [45,46]. On the other side, immortalization of human granulosa cells from oocyte retrieval during IVF (HGL5 and HO-23) resulted in cells lacking responsiveness to FSH or human chorionic gonadotropin (hCG) [47,48]. Bayasula et al.’s work on nonluteinized granulosa cell immortalization [49] reports a very similar transcriptome of lentivirus immortalized HGrC1 when compared with primary granulosa cells. Unlike other granulosa cell models, HGrC1 show proliferation and steroidogenesis under gonadotropin regulation, suggesting that this model keeps original granulosa cell functional activity.

As discussed for SCs, animals can be also a source of GCs. In the same way as SCs models, species-specific differences in granulosa cell function make these models not ideal for human mechanism studies. As an example of this hypothesis, it is known that mutations of the bone morphogenetic protein 15 (*BMP-15*) gene cause infertility in sheep and POF in humans while mice show normal follicle development [50,51,52].

### 2.3. Germ Cell Models

Modelling germ cell lineage can add an extra layer of difficulty considering that they are the only cells transmitted to the next generation. Germ cells are tightly dependent on the interaction of multiple cell types for their development and differentiation [53]. The mechanisms underlying primordial germ cell (PGC) specification are not conserved even across related species. In mammals, recent studies suggest that the specification of mice PGCs differs from those in humans, suggesting that different molecular regulatory mechanisms govern PGC development in both species [54,55,56,57]. Therefore, researchers look at embryonic stem cells (ESCs) for alternatives. Many groups have successfully differentiated germline cells from ESCs in vitro [58,59,60,61,62]. These cells, known as primordial germ cell-like cells (PGCLCs), show a great potential for the study of germ cell differentiation and their interaction with somatic precursors during sexual development. However, embryonic stem cell research depends exclusively on ESCs lines isolated from “discarded” embryos in IVF clinics. This is difficult to reconcile with the need to obtain patient-specific cell sources for the study of DSD.

## 3. Unleashing the Power of Pluripotency: iPSCs and Guided Differentiation

**Figure 2:**
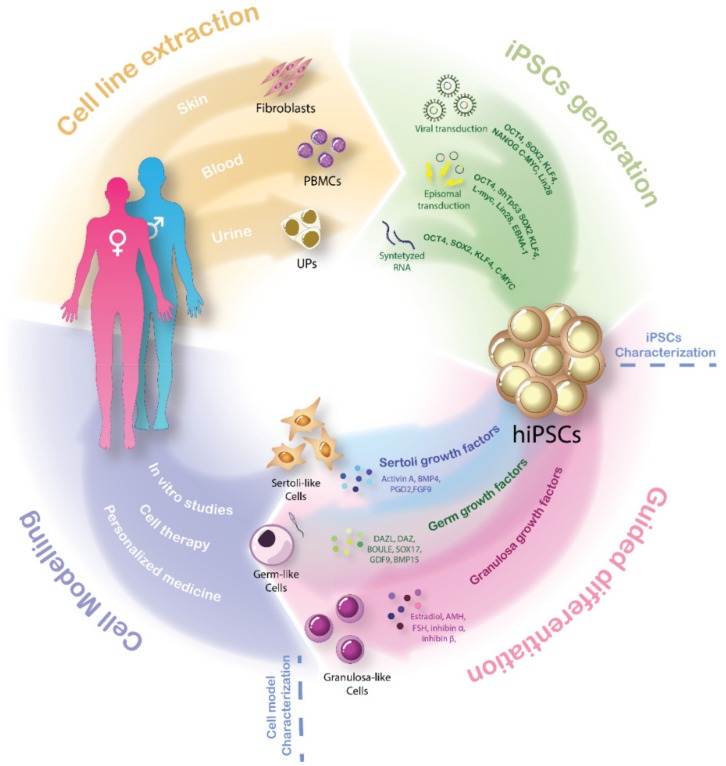
Cellular reprograming and guided differentiation of hiPSCs: The scheme represents the four stages of the iPSCs-derived gonadal cell model generation for DSD patients. Isolation of fibroblasts, peripheral blood mononuclear cells or even urinary precursors directly from the patient have been considered as easy to obtain and patient-friendly cell sources. Up to date, three cellular reprogramming techniques are commonly used to generate hiPSCs from diverse cell sources: viral transduction of Yamanaka and Thomson reprograming factors; episomal transduction adding a EBNA1 factor and silencing expression of the *TP53* gene; and synthetized RNA. Guided differentiation by expression specific factors can force transformation of cell into Sertoli-like (BMP4, Activin A, PDG2 and FGF9) Granulosa-like (Estradiol, AMH, FSH, inhibin α and inhibin β) or germ-like cells (DAZL, DAZ, BOULE, GDF9 and BMP15). Addition of both those factors into the culture medium and forced overexpression proved to induce differentiation of hiPSCs. Gonadal cell-like models directly derived from DSD patients suppose unique tools to perform patient-specific in vitro studies and open the door for more advances in cell therapy and personalized medicine for DSD patients. hiPSCs: human induced pluripotent stem cells, PBMCS: peripheral blood mononuclear cells, UPS: urinary progenitors.

The availability of specific cell models for the diverse DSD variants might help to better understand the mechanisms involved in human sex development and to improve care for DSD patients. New technologies in the area of cell reprogramming opened a window of opportunity for a revolutionary form of personalized medicine: the reprogramming of terminally differentiated cells into induced pluripotent stem cells (iPSCs) and their guided differentiation into virtually any cell type (Figure 2).

In 2006, Shinya Yamanaka’s group combined the previous knowledge on nuclear transfer [63,64,65], transcription factors [66,67,68] and embryonic stem cells [69,70,71] to induce reprogramming of terminally differentiated fibroblasts into pluripotent stem cells by retroviral transduction [72]. From 24 different factors tested, Yamanaka et al. discovered that the combination of POU class 5 homeobox 1 (OCT3/4), kruppel like factor 4 (Klf4), SRY-box 2 (SOX2), and proto-oncogene C-Myc (C-Myc), all related to undifferentiated ESCs self-renewal and maintenance, played a key role for the generation of the iPSCs. Thanks to this pioneering work, Yamanaka was awarded the Nobel Prize in Physiology or Medicine 2012.

Although iPSCs maintain the essential features of ES cells to propagate in culture indefinitely and to differentiate into each of the three embryonic germ layers, their origin from somatic cells and the reprogramming methods used may lead to significant differences between iPSCs and ESCs [73]. Additionally, the use of retrovirus in the mentioned Yamanaka study to transduce reprogramming factors has certain limitations in personalized medicine due to observed integrations of those factors into the endogenous genome [72]. Furthermore, *C-MYC* is a known prooncogene and its function as a proliferation enhancer may be achievable by other nononcogenic factors, resulting in less tumorigenic iPSCs [74,75]. Yu et al. [76] refined the reprogramming procedure adding a different cocktail of factors. In this case, *OCT4*), *SOX2*, nanog homeobox (*NANOG*), and lin-28 homolog A (*LIN28*) were transduced using lentivirus, which increased the efficiency of reprogramming due to the ability to infect non dividing cells. Although of great potential for general cell models, the use of lentivirus transduction is also not very suitable for patient-specific modeling since, despite reducing the carcinogenic potential of *c-MYC*, the insertional mutagenesis is still a concern [77].

During recent years, many researchers sought alternative ways to generate integration-free iPSCs. Episomal vectors [78], synthesized RNA [79] and Sendai virus, a single chain RNA virus [80], have become of routine use in reprograming experiments. Every system shows advantages and disadvantages that may be differently adapted for every laboratory. Technology based on RNA like synthetized mRNA or Sendai virus completely eliminate the risk of genomic integration intrinsic to all DNA-based approaches but modified mRNA is technically complex and Sendai virus experiments need to be performed under strict biological containment [80]. Conversely, episomal vectors offer an excellent approach due to the simplicity and reproducibility of the method [81]. Modified-mRNAs are lost extremely quickly from host cells and this reprogramming methodology requires daily transfections [82]. Both episomal vectors and Sendai virus remain in the cytoplasm of the host cells but they are lost after several generations [83].

As to the intrinsic efficiency of every iPSCs generation methodology, we have to add the fact that not all cell types are equally receptive to reprogramming [84]. Moreover, scientists have recognized important differences between iPSC lines from diverse origins. These variations are mostly connected to the inherent plasticity of somatic cells [85,86,87]. Novel insights suggest that the original metabolic environment affects the faith of future iPSCs even after reprogramming [88,89]. This principle seems to also affect SC-derived iPSCs [90]. Epigenetic memory was gradually lost in iPSCs after passages, due to either post-reprogramming removal of the epigenetic pattern of somatic cells or selective pressure against iPSC with epigenetic memory [89]. Fibroblasts are the most common cell source used for iPSCs generation thanks to good reprogramming efficiency rates and the simplicity of their culture. Nevertheless, peripheral mononuclear blood cells (PBMCs) [91] or urinary progenitors (UPs) collected from spontaneous urine [92,93] have gained importance in an effort to obtain a high amount of reprogrammable cell sources with a less invasive sampling procedure.

Developing new iPSCs requires exhaustive characterization of new lines in order to define the iPSC populations and to demonstrate their pluripotency. The analysis of the endogenous expression of pluripotency markers like OCT4, stage-specific embryonic antigen 3 (SSEA3), stage-specific embryonic antigen 4 (SSEA4) and TRA-1-81 allows for discrimination between high quality iPSCs and partially reprogrammed colonies. The prolonged culture of iPSCs lines can result in genetic abnormalities causing aneuploidy. Thus, analysis of the karyotype of iPSCs is of vital importance when aiming to provide patient-specific medicine [94]. iPSCs colonies that show genetic abnormalities must be discarded to preserve resemblance to the patient. In addition, high quality iPSCs must demonstrate their ability to differentiate into primordial germ layers of the embryo, i.e., ectoderm, mesoderm and endoderm, in vitro and in vivo [95].

Once stablished, an iPSCs cell line has the potential to differentiate in multiple cellular lines. This process may occur spontaneously, causing the contamination of the culture with undesired populations. A tight control on the environment is necessary to force differentiation of all cells through the same desired path. The first step involves the generation of embryoid bodies, wherein differentiation occurs as a result of the transfer of iPSCs into a suspension culture environment. From this point on, a tight differentiation control can be achieved by the step-wise addition of growth factors, cytokines or inhibitors based on the knowledge of specific differentiation pathways for every desire cell line [96].

## 4. Modeling the Complexity: Human iPSCs-Derived Models for DSD Research

Our knowledge of human sex differentiation has increased progressively in recent years although advances were not equally represented in both genders. Mechanisms underlying male sexual differentiation have been well studied while many factors regulating female sexual differentiation remain unknown. The current gonadal cell models generated through human iPSCs (hiPSCs) differentiation are summarized in Table 1.

### 4.1. hiPSCs and Somatic Gonadal Cell Models

In SCs modelling, better understanding of many of the factors involved finally offered us the opportunity to recapitulate somatic support cell differentiation in vitro in a relatively accurate manner. In this respect, original work by Buganim et al. proved the ability of iPSCs to differentiate into SCs [109] in mice. Bucai et al. [97] observed that, when human embrionic stem cells (hESCs) are differentiated into PGCs, the same PGCs seem to guide the differentiation of uncommitted or primitive cells present in the culture into Sertoli-like cells (SLCs), possibly through paracrine factors released in the medium. After obtaining similar results with umbilical cord perivascular cells, Shlush et al. [98] identified the secretion of bone morphogenic protein 4 (BMP4) by undifferentiated cells to be pivotal for the differentiation into SCs.

SOX9, fibroblast growth factor basic (bFGF), fibroblast growth factor 9 (FGF9), prostaglandin 2 (PGD2) and Steroidogenic Factor 1 (SF1) have also been considered as candidate factors due to their role in Sertoli cell differentiation. SOX9 expression is crucial in SCs differentiation and needs to be maintained during the whole process. bFGF and BMP4 are two key regulators in the formation of the urogenital system, via mesoendoderm signaling [110,111]. It is known that both factors are able to stimulate the expression of SOX9 [112]. The expression of SOX9 can also be stimulated in an independent way by PGD2 and FGF9 [113,114]. Remarkably, the action of SF1 has been emphasized as essential for fetal SCs survival. SF1 controls the cell cycle of Sertoli cells during differentiation by regulating the tumor protein P53 (TP53) pathway [115] while up to date, to our knowledge no peer-reviewed articles have proved its effect on iPSCs guided differentiation.

In our study [37], embryoid bodies were treated with a cocktail of growth factors including bFGF, BMP4, PGD2, FGF9 and activin A to promote the endogenous expression of SOX9 until the cells changed morphology and resembled SCs appearance. The elevated expression of SOX9 observed reflected its successful stimulation by the administered growth factors cocktail. The addition of activin A to the cocktail allowed the further expansion of the culture due to its role in SCs proliferation [116].

Characterization with new generation sequencing techniques revealed that this was new and until today the unique human SLCs model is actually closer to HSerCs than the cancer-derived NT2d1 cells. Indeed, SLCs bear a greater resemblance to fetal SCs than to mature cells, a characteristic observed in other iPSCs-derived models [96], but with great interest for the study of SC role during sexual development. These SLCs are still a proof of concept and have some limitations including the use of lentivirus transduction to generate iPSCs and the lack of a long-term study of SLCs characteristics. Nevertheless, it opens the way to patient-specific SC models in DSD research.

Several successes were also achieved in GC modeling. Experiments with murine cells [117,118] demonstrate the effective differentiation of mouse and rat iPSCs into GC-like cells by co-culture with GCs. Those GC-like cells expressed several genes related to granulosa cell function like BMP15, FSHR, AMH and GDF9 and were able to secrete estrogen. These observations highlight the fact that cell-cell connection and factors generated by the differentiated GC in the co-culture can induce the differentiation of stem cells through the granulosa pathway. In line with these results, Liu et al. in 2016 [100] induced the differentiation of human iPSCs into GC-like cells through the addition of a growth factors cocktail to the culture. The cocktail was composed by all-trans-retinoic acid, estradiol, AMH, FSH, Inhibin α, Inhibin β and TGF–β; all crucial for the maintenance of ovarian follicular development and secreted by GCs.

In an independent way, Lipskind et al. [101] demonstrated that native differentiation of human amniocytes-derived iPSCs can produce functional granulosa-like cells without intervention of additional growth factors. Whether this approach is valid for iPSCs derived from different cellular origins has to be elucidated. This growth factors-free differentiation might be taking place thanks to the metabolic memory discussed above, turning the differentiation balance into GCs faith. In line with these thoughts, Anchan et al. [99] have shown how iPSCs derived from GCs were able to redifferentiate into homotypic ovarian steroidogenic cells in vitro. Even though the results of this methodology are inapplicable for most of DSD conditions, it pointed out that the quality of the iPSCs-derived cell model may be dependent on the original cell source.

### 4.2. hiPSCs and Germ Cell Models

The accurate timing of human germ cells specification cannot be determined precisely due to the difficulties and ethical boundaries of using human embryos for research studies. We already mentioned that the mechanisms leading to germ cell specification are not well conserved among different species, making the previous differentiation approaches in mouse and rat iPSCs [119,120] not completely applicable to human cell models [121]. In humans, SOX17 seems to have a key role in embryonic germ cell differentiation and controls the expression of B-lymphocyte-induced maturation protein 1 (BLIMP1), which is the leading gene of germ cell induction in mice. Additionally, SOX2 is essential for mouse germ cell development but is not detected in human embryonic germ cells [55,122]. There are, however, some islands of germ cell differentiation mechanisms that are well conserved across the animal phyla, specially related to mitotic and meiotic phases of germ cells development [123,124]. Those common genes may be used as a foundation stone to build our knowledge of human germ cell development. For instance, genes like nanos homolog 3 (*NANOS3*) that proved to be crucial during murine germ cell development, seem to play a maintenance role in early germ cell development in humans [125,126].

In the quest to unravel the machinery of germ cell specification and development in both sexes, first attempts from Kee et al. [127] reported the importance of bone morphogenetic protein (*BMPs*) genes to promote germ cell formation from iPSCs. The same group discovered the action of deleted in azoospermia like (DAZL), deleted in azoospermia (DAZ) and boule homolog (BOULE) promoting germ-cell progression to meiosis and formation of haploid germ cells [102]. They also demonstrated that the expression of vasa homolog (VASA), a germ cell-specific marker, is indeed regulated by DAZL. The DAZ family, located on the Y-chromosome, is one of the conserved clusters among species and has been reported as essential for male fertility. Its deletion leads to azoospermia and oligospermia in humans. These advances allowed for the transformation of iPSCs even from adult human cell sources into advanced meiotic germ cells by overexpression of DAZL, DAZ and BOULE proteins [104,106]. Alternatively, hiPSCs-derived robust meiotic germ cells were obtained by a two-step protocol, removing bFGF at first and subsequently RA, forskolin, leukemia inhibiting factor (LIF) and CYP26 inhibitor [103]. hiPSCs differentiated with mouse spermatogonial stem cell medium expressed markers for post-meiotic spermatocytes and round spermatids [107]. Nonetheless, in our knowledge, complete spermatogenesis in vitro has not yet been accomplished in human.

In human female iPSCs, Eguizabal et al. showed that overexpression of DAZL and BOULE can also lead iPSCs to enter meiosis. Yang et al. [105] used fibroblasts from premature ovarian insufficiency (POI) patients to successfully differentiate them into PGCs that had the potential for meiotic progression in vitro. Mimicking the natural role of the oocyte by stimulation with GDF9 and BMP15 proved further induction of folliculogenesis in differentiated PGCs [108]. Transcriptome analysis of these follicle-like cells (FLCs) resembled in vivo primordial follicles. In the same study, researchers observed that the population of differentiated ESCs organize itself when exposed to the differentiation factors, generating an oocyte-like cell surrounded by a cellular aggregate with Granulosa-like cells at the outer layer. Together with the previous observations discussed above, this new evidence brings increased consistency to the hypothesis of microenvironment guidance over GCs differentiation.

**Figure 3:**
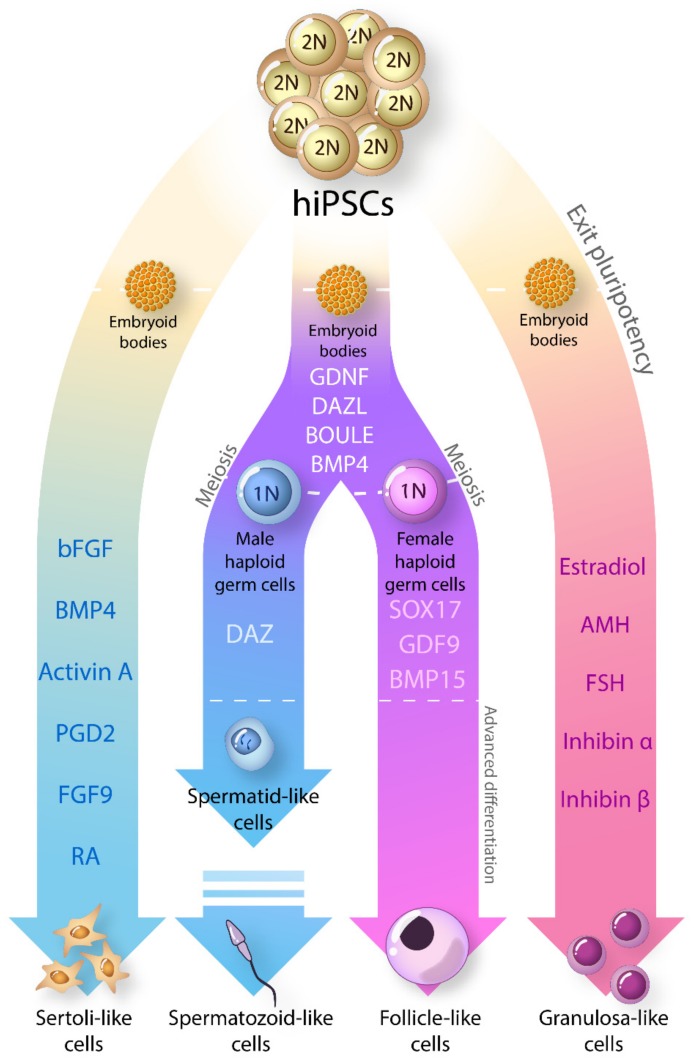
Known factors involved in guided differentiation of hiPSCs into gonadal cells: Expression of growth factors can guide the differentiation of embryoids bodies into Sertoli, Granulosa or germ cell-like cells. Bone morphogenic protein 4 (BMP4), Activin A, prostaglandin 2 (PDG2) and fibroblast growth factor 9 (FGF9) and retinoid acid (RA) successfully differentiated into Sertoli-like cells are able to endogenously express SOX9, anti-Mullerian hormone (AMH), claudin-11 (CLDN11) and other Sertoli markers [37,98]. Granulosa-like cell differentiation has shown to be stimulated by estradiol, AMH, follicle stimulating hormone (FSH), inhibin α and inhibin β [109,110,111]. BMPs proteins together with GDNF, DAZL and BOULE are known to initiate differentiation into a meiotic primordial germ cell-like (PGCL) phenotype [55,102,103,104]. While complete spermatogenesis in human cells was not achieved yet, PGCs differentiated with SOX17, GDF9 and BMP15 factors showed the ability to induce folliculogenesis [108].

Taken together, great progress has been accomplished in gonadal cell modeling, proving that both male and female iPSCs enclose the potential to differentiate into any gonadal cell type, including germ cells (Figure 3). The use of cellular reprogramming is now leading a pioneering pathway in the generation of fully patient-specific cell models with enormous advantages for their use in DSD research.

## 5. Future of DSD Models

Our comprehension of the complex processes that drive the mechanism of disease in DSD patients has always been limited by the lack of the proper cells involved in those mechanisms, either by the impossibility to obtain samples from the patient or the absence of orthologous cellular models.

For the last 12 years, the innovative techniques of cellular reprogramming has sparked a revolution in many areas of medicine, including DSD research. Thanks to this powerful tool, we are now able to reprogram virtually any cell form a patient and iPSCs have become an exceptional autologous cell source for cell replacement therapy for a large number of diseases. Looking at DSD patients, we have the opportunity to guide iPSCs re-differentiation into somatic supporters and germ cells, making personalized cell models to reveal the individual complexity underlying such a diverse group of conditions.

Nevertheless, the capacity of differentiation techniques to guide correctly the destiny of the cells is completely dependent on our knowledge of the processes governing the sexual development in humans, which are not totally understood.

### Limitations and Uncertainties

Cellular models resembling early development Sertoli and granulosa cells as well as primordial germ cells lacked long-term stability. For example, our preliminary results of long-term culture of SLCs showed a reduction of SOX9 expression after 30 days of culture. This time window suffices to study the mechanism of disease in DSD patients but might be too short for more complex long-term studies, such as therapy or regenerative medicine.

One major challenge that remains is the difficulty to generate terminally differentiated cells from iPSCs. As we discussed through this review, cell differentiation is a highly complex process probably including not only gene transcription changes but also epigenetic and translational regulations that may be essential for late differentiation steps. Taking advantage of the astonishing evolution of new generation techniques, we can now explore the effects that epigenetic and proteomic regulations may have on cellular reprogramming and differentiation. Proteomic study in iPSCs-derived hepatocytes [128] has identified critical regulatory molecules that creates a transcriptional fingerprint during terminal differentiation. We are confident that further studies will provide additional clues to understand the implication of posttranslational regulation in the terminal differentiation of iPSCs-derived cultures, including gonadal cell models.

Novel techniques in gene editing can be also translated into iPSCs-cell modeling research. CRISPR/Cas9 has proven to be a versatile genetic editing tool to correct mutations on iPSCs-derived cell models [129]. Researchers can now study modifications in disease severity by introducing gene deletion or repairing knocked-out expression in isogenic iPSCs-derived cells [130].

A second point to discuss is the selection of cellular sources. As we already mentioned [99,101], the pre-iPSC origin has a strong influence on the faith of (re)differentiated cells. Cellular populations from tissues with ontogenetic similarities to gonadal structures necessitate less complex guided differentiation to generate gonadal somatic supporters. By selecting original cell sources closely related to gonadal cells, we may increase the quality of future cell models. The combination of both a better understanding of ruling factors of sex development and selection of the best cell source available would produce cell models of excellent quality with greater stability under long-term culture.

Another factor that has gained importance is the physical and chemical communication between all the cells of a niche for their correct differentiation and maintenance [39,42,117,118]. This concept is not new: in adult tissues, regeneration is maintained by populations of multipotent progenitor cells that keep proliferation abilities and differentiate when needed. This differentiation is regulated by signals flowing from terminally differentiated cells of the niche to fulfill the demands of the tissue [131]. This idea of the microenvironment as a driving force for differentiation includes not only cell-cell interactions but also soluble factors, extracellular matrix (ECM) proteins and physical conditions like hypoxia, pressure or stiffness of the culture surface [132]. The generation of a synthetic microenvironment that mimics the physiologic conditions where Sertoli, granulosa and germ cells are developed might boost the efficiency of the differentiation process by reducing the chances of spontaneous differentiation and increasing the stability of the whole complex. To this respect, it is now possible to coordinate stem cells into 3D structures, known as organoids, which resemble the native multicellular architecture and functional features of a broad range of organ tissues [133]. Organoids and other 3D-models like organs on a chip (OOC) are platforms to understand the unique mechanisms of human developmental biology and offer an opportunity to model diseases in a more complex way, including those affecting sexual development.

We envision that co-culture of Sertoli-like cells and spermatid-like cells derived from the same cell source within a synthetic matrix that recreates the microtubules environment may not only improve the stability of both cell models but also have a synergistic effect, where SLCs would be able to mature and promote spermatogenesis. This is equally applicable to oocytes, surrounded by granulosa-like cells in a synthetic scaffold as suggested by Laronda et al. [134]. The authors also hypothesized that triggering the initial steps of development and maturation by FSH and LH in oocytes and by RA for Sertoli cells [135], these synthetic ovary or seminiferous tubules would mimic the natural release of all hormones and factors necessary to support reproductive features [134]. Woodruff’s group developed a two-step follicle culture strategy, supplementing medium with E2, FSH and human growth hormone (hGH) followed by the addition of AMH and estradiol, as a recapitulation of the dynamic human follicle growth environment [136]. A distinct advantage of using iPSCs for this purpose lies on their patient-specific origin so that their antigenically match to the acceptor cells, provide a platform for autologous therapies. Applied to a co-culture of hiPSCs-derived cell models, this methodology could improve the (re)generation of personalized synthetics reproductive organs.

The works reviewed are still mainly a proof of concept but lay the groundwork for what would come in the following years. The standardization of both every-time better reprograming methodologies and new generation analyses will aim to boost the quality and robustness of iPSCs-derived models. Together with an increased knowledge of the mechanisms and players involved in gonadal cells differentiation, these advances would open the door for truly patient specific gonadal cell models of priceless value to understand the mechanism of disease in different DSD conditions.

The production of gonadal cells from DSD patients may enable not only the of study in vitro therapies that target the factors affected in DSD but also boost our understanding of the key elements behind normal sex development and gonadal cell survival. This may ultimately contribute to new strategies for the diagnosis and treatment of unexplained infertility, a common health problem that affects approximately 15% to 30% of couples at reproductive age [137].

## Figures and Tables

**Table 1:** Human PSCs-Derived Gonadal Cell Models.

Cell Type	Cell Model	Differentiation Mechanism	Remarks	Reference
Sertoli cell	SLC	Unguided differentiation in co-culture with PGCs	Possible paracrine action of PGCs on differentiation of SLCs	Bucay et al., 2009 [97]
	SLC	5-step differentiation protocol, including RA, LIF, GDNF, putrescine, testosterone and FSH	BMP4 secretion by undifferentiated cells	Shlush et al., 2017 [98]
	SLC	Addition of BMP4, PGDS, bFGF, FGF9 and Activin-A to growth medium	Transcriptomic landscape resemble differentiating Sertoli cells	Rodríguez Gutiérrez et al., 2018 [37]
Granulosa cell	Ovarian steroidogenic cells	Redifferentiation of iPSCs derived from GCs into homotypic ovarian steroidogenic cells	Cellular origin have strong effect on final differentiation faith	Anchan et al., 2015 [99]
	GLC	Addition of all-trans-retinoic acid, estradiol, AMH, FSH, Inhibin α, Inhibin β and TGF –β to growth medium	GLCs can rescue ovarian failure when transplanted in POF mice.	Liu et al., 2016 [100]
	GLC	Differentiation from human amniocytes without intervention of additional growth factors.	GLC able to synthetize E2	Lipskind et al., 2018 [101]
Primordial germ cell	hPGCLCs	Differentiation induction by recombinant BMPs	BMPs induce differentiation of germ cells from hES cells	Kee et al., 2009 [102]
	Haploid Gamete-like Cells	Two step protocol: Culture in bFGF-depleted ES cell media followed by addition of RA	Epigenetic memory of the reprogrammed somatic cells	Eguizabal et al.,2011 [103]
	Meiotic GCs	Overexpression of VASA and/or DAZL following differentiation on matrigel-coated plates	Differentiation of germ cells is dependent on post-translational regulation by RNA-binding proteins like VASA	Medrano et al., 2011 [104]
	hPGCLCs	Addition of BMP2 or BMP4, LIF, SCF, EGF and ROCK inhibitor to growth medium	SOX17 is the key regulator of hPGCLC specification	Irie et al., 2015 [55]
	hPGCLCs	(1) in vitro: same protocol as Irie et al., 2015 (2) in vivo: xenotransplantation into NOD/SCID mice	hPGCLCs had the potential for meiotic progression in vitro	Yang et al., 2019 [105]
Spermatid	SpLC	Differentiation induction by BMPs followed by overexpression of DAZ, DAZL, and BOULE	hiPSC lines can differentiate to haploid cells with characteristic staining of ACROSIN for spermatid.	Panula et al., 2011 [106]
	SSC	Direct differentiation using mouse spermatogonial stem cell (SSC) medium	hPSCs differentiate into spermatogonia, spermatocytes and haploid spermatids	Easley et al., 2012 [107]
Oocyte	FLCs	Overexpression of DAZL and BOULE with recombinant human GDF9 and BMP15	GDF9 and BMP15 induce ovarian follicle formation in hESCs	Jung et al.,2017 [108]

FLCs: follicle-like cells, GLC: Granulosa-like cells, hPGCLCs: human primordial germ cell-like cells, SLC: Sertoli-like cells, SpLC: spermatid-like cells, SSC: spermatogenic stem cells.
